# Type 2 diabetes as a disease of ectopic fat?

**DOI:** 10.1186/s12916-014-0123-4

**Published:** 2014-08-26

**Authors:** Naveed Sattar, Jason MR Gill

**Affiliations:** Institute of Cardiovascular and Medical Sciences, BHF Glasgow Cardiovascular Research Centre, University of Glasgow, 126 University Place, Glasgow, G12 8TA UK

**Keywords:** Insulin resistance, NAFLD, Pancreas, Adiposity, Sex, Ethnicity, Family history of diabetes

## Abstract

**Background:**

Although obesity and diabetes commonly co-exist, the evidence base to support obesity as the major driver of type 2 diabetes mellitus (T2DM), and the mechanisms by which this occurs, are now better appreciated.

**Discussion:**

This review briefly examines several sources of evidence – epidemiological, genetic, molecular, and clinical trial – to support obesity being a causal risk factor for T2DM. It also summarises the ectopic fat hypothesis for this condition, and lists several pieces of evidence to support this concept, extending from rare conditions and drug effects to sex- and ethnicity-related differences in T2DM prevalence. Ectopic liver fat is the best-studied example of ectopic fat, but more research on pancreatic fat as a potential cause of β-cell dysfunction seems warranted. This ectopic fat concept, in turn, broadly fits with the observation that individuals of similar ages can develop diabetes at markedly different body mass indexes (BMIs). Those with risk factors leading to more rapid ectopic fat gain – for example, men (compared with women), certain ethnicities, and potentially those with a family history of diabetes, as well as others with genes linked to a reduced subcutaneous adiposity – are more likely to develop diabetes at a younger age and/or lower BMI than those without.

**Summary:**

Obesity is the major risk factor for T2DM and appears to drive tissue insulin resistance in part via gain of ectopic fat, with the best-studied organ being the liver. However, ectopic fat in the pancreas may contribute to β-cell dysfunction. In line with this observation, rapid resolution of diabetes linked to a preferential and rapid reduction in liver fat has been noted with significant caloric reduction. Whether these observations can help develop better cost-effective and sustainable lifestyle /medical interventions in patients with T2DM requires further study.

## Background

Although obesity and type 2 diabetes mellitus (T2DM) commonly co-exist, the evidence to show that obesity is a cause of T2DM, and the mechanism by which it does so are only now beginning to be fully appreciated. Here, we discuss the range of evidence to support this view. We also describe the concept of ectopic fat as an important mechanistic link between obesity and T2DM in many individuals.

## Discussion

### Epidemiological associations

**Figure 1:**
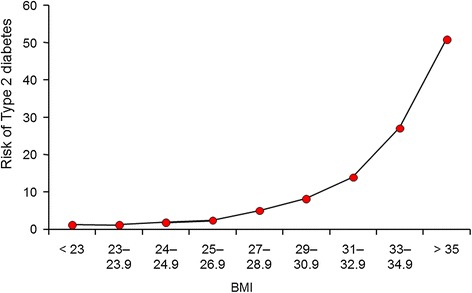
**Relationship between body mass index (BMI) and risk for diabetes in US Health Professionals, derived from data extracted from Chan** ***et al.*** [2]**.**

The strong epidemiological link between obesity and T2DM in general is, of course, well described [1]. The relative risks (RRs) for diabetes with rising obesity are in fact among the highest for any risk factors for any disease, with around a 50- to 80-fold increase in T2DM risk for a BMI of over 35 kg/m^2^ compared with a BMI of less than 23 kg/m^2^ in populations of white European descent (Figure 1) [2]. As a result, BMI can account for a third to half of the weighting for T2DM risk scores. Thus, the increasing level of obesity is a key driver for the rising T2DM levels around the world, alongside improved survival of patients with diabetes due to lower death rates, and rising life expectancy in general. Unsurprisingly, therefore, any simple measure of adiposity alone (for example BMI, waist circumference or waist/hip ratios) can predict incident T2DM, with area under the curve (AUC) for receiver operator characteristic (ROC) ranging from 0.66 to 0.73 [3].

### Genes strengthen causal links between obesity and diabetes

The most important gene for obesity so far discovered, *FTO* (fat mass and obesity-associated protein), was first reported to be linked to T2DM by Frayling *et al*. in 2007 [4]. Approximately 16% of Europeans are homozygous for high-risk variants of the *FTO* gene, and this is associated with an average body weight 3 to 4 kg greater than normal. However, the higher risk for T2DM in Europeans is completely abolished after adjusting for BMI, suggesting that the risk conferred by *FTO* is mediated via higher body mass in this ethnic group, although other data suggest that in other ethnicities, *FTO* may influence T2DM risk in part independent of BMI [5]. Multiple other genes are linked to T2DM risk, most being linked to β-cell dysfunction rather than insulin resistance, as reviewed by Loos and Bouchard [6]. In terms of T2DM risk assessment, however, there is no benefit in genotyping individuals for their T2DM genes because such information does not improve upon the risk prediction achievable by using a simpler clinical phenotype and knowledge of family history of T2DM [7]. Nevertheless, continued work on T2DM-related genes should help strengthen causal inferences on new risk pathways.

### Evidence for the ectopic fat hypothesis in T2DM

Surprisingly, the pathophysiological basis for T2DM continues to attract debate. What is clear, however, is the emergence of one major hypothesis, namely that of ectopic fat leading to organ-specific insulin resistance via a process termed ‘lipotoxicity’ [8]. Many individuals prone to T2DM appear to show a greater propensity to accumulate visceral fat for a given weight; interestingly, this characteristic may be a downstream consequence of an ‘impaired’ subcutaneous fat storage capacity, the mechanisms for which deserve greater attention. As extreme example of this concept is lipodystropy; individuals with this condition have an impaired ability to store subcutaneous fat, and consequently with even modest weight gain, they accumulate fat in visceral and ectopic tissues (for example, the liver), leading in turn to marked insulin resistance [9]. At the other extreme are many individuals, particularly women, who, despite attaining very high BMIs (as high as 50 to 60 kg/m^2^), remain insulin-sensitive, normoglycaemic and normolipaemic. Imaging studies show these individuals to have low levels of visceral and ectopic fat but a high subcutaneous fat content [10]. A pharmacological example underlying the importance of ectopic fat to dysglycaemia comes from the observation that peroxisome proliferator-activated receptor (PPAR)-γ agonists (thiazolidenediones) lower glucose levels despite increasing the patient’s weight. They appear to do so by redistributing fat away from the liver and towards an expanded subcutaneous pool [11,12]. Finally, transplantation of normal adipose tissue in the subcutaneous region of lipoatrophic mice, which are normally severely insulin-resistant, removes their excess hepatic fat and normalises hepatic insulin sensitivity [13].

### Ethnicity, sex, and the ectopic fat hypothesis

There are substantial ethnic differences in diabetes risk. For example, South Asians develop T2DM at lower BMI levels and around a decade earlier in life than white Europeans [14]. Using UK Biobank data, we have recently shown that South Asians with BMI of 22 kg/m^2^ have equivalent prevalence of T2DM to white Europeans with BMI of 30 kg/m^2^. The corresponding BMIs for Chinese men and women are 26 and 24 kg/m^2^, respectively [15]. These remarkable findings help to explain why T2DM rates are rapidly escalating as obesity levels rise in countries such as India and China. A key reason for the greater increase in diabetes risk per unit increase in BMI in South Asians compared with Europeans may be due to a reduced capacity in South Asians to store fat in the primary superficial subcutaneous adipose tissue compartment, leading to earlier ‘overflow’ into secondary deep subcutaneous and visceral fat compartments, and potentially the liver [16]. Indeed, there is some evidence of differences in morphology of subcutaneous abdominal adipose cells in South Asians compared with Europeans, consistent with reduced capacity to store fat in this depot [17–19], and other evidence suggests that South Asians may have higher levels of liver fat than Europeans [19,20]. Current evidence on ethnicity and ectopic fat is limited by its cross-sectional nature, and further research evaluating ethnic differences in the longitudinal changes in subcutaneous fat storage, adipocyte morphology and function, and ectopic fat deposition with increasing adiposity may yield important insights into the mechanisms underpinning the substantial ethnic differences in diabetes risk.

In terms of sex, we recently showed that men develop diabetes at lower average BMIs compared with women at most adult ages [21]. This finding is consistent with the observation that adult men have greater levels of liver fat and insulin resistance than women of comparable BMI [22]. In general, women carry or have a greater subcutaneous fat storage capacity and, linked to this, carry less visceral fat than do men. Therefore, the average woman has to put on more weight than the average man to reach the point at which she overwhelms her subcutaneous stores and promotes sufficient ectopic fat deposition to develop hyperglycaemia.

**Figure 2:**
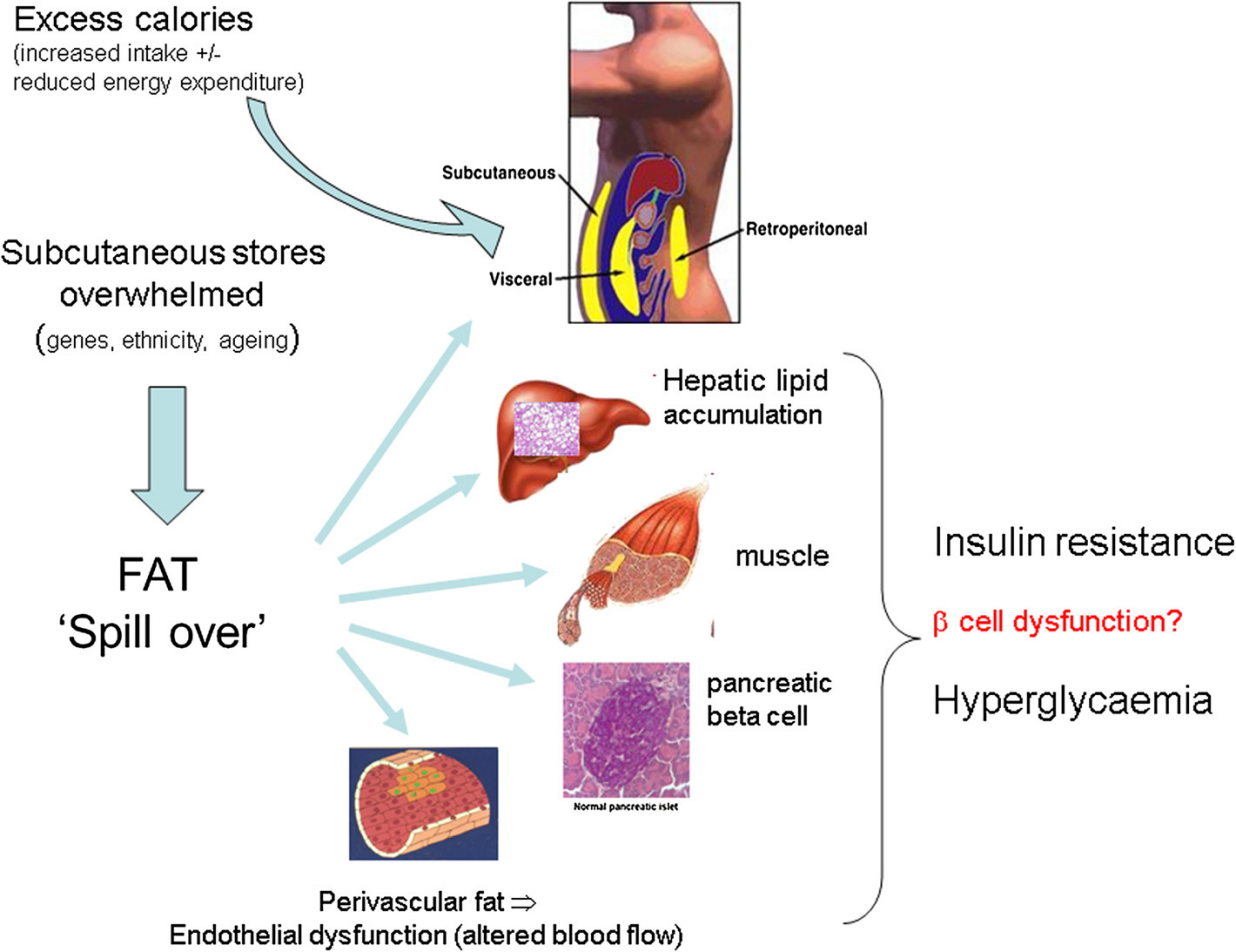
**Simple concept of ectopic fat and development of insulin resistance and frank diabetes.** A simple conceptual illustration on the development and location of ectopic fat in individuals once they have ‘overwhelmed’ their ability to store safe subcutaneous fat. Certain factors such as sex (females have greater storage capacity), genetics (with family history of type 2 diabetes mellitus (T2DM) as a broad proxy measure), ethnicity (for example, South Asians) and ageing appear to have relevance to an individual’s ability to store fat subcutaneously. Other factors, such as smoking, may also be relevant but more data are needed to examine this. In temporal terms, it may be that liver fat accumulation occurs closer to the time of development of T2DM whereas muscle insulin resistance is a more proximal development. Perivascular fat may contribute to vascular dysfunction via a process of adverse vasocrine signalling, leading in turn to impaired nutrient blood flow; that is, vascular insulin resistance. Finally, some recent evidence indicates that excess fat may also accumulate in the pancreas to contribute to β-cell dysfunction, and thus development of T2DM. Such excess pancreatic fat appears reversible, and could contribute to diabetes resolution even in some patients with T2DM who are on insulin.

Collectively, the foregoing observations suggest that the preferential location of fat storage (subcutaneous versus visceral/ectopic) at any given BMI in individuals may be crucial to their risk of a metabolic disorder, inclusive of diabetes. Figure 2 summarises the concept of ectopic fat.

As an example showing that differential BMI thresholds depend upon several genetic factors, a 50 year-old South Asian man with a strong family history of T2DM has a near-equivalent 10 year risk (~13% QDdiabetes risk score [23]) of T2DM at a BMI of 22 kg/m^2^) as an age-matched white female with no family history of T2DM with a BMI of nearly 40 kg/m^2^. Thus, sex and ethnicity combine to profoundly influence diabetes risk, and this is likely to reflect, in part, differences in propensity for ectopic fat storage, although of course, other factors also contribute.

### The crucial role of ectopic liver fat and related molecular explanations

In terms of chronology, gain of liver fat appears to precede development of T2DM in most individuals, whereas muscle insulin resistance appears to be a longer-standing and earlier abnormality [24]. Considerable recent work has focused on the role of the liver in the development of diabetes, and linked to this, the condition of non-alcoholic fatty liver disease (NAFLD) has been shown to be very common (>50%) in patients with T2DM, and is linked to hepatic insulin resistance [25].

**Figure 3:**
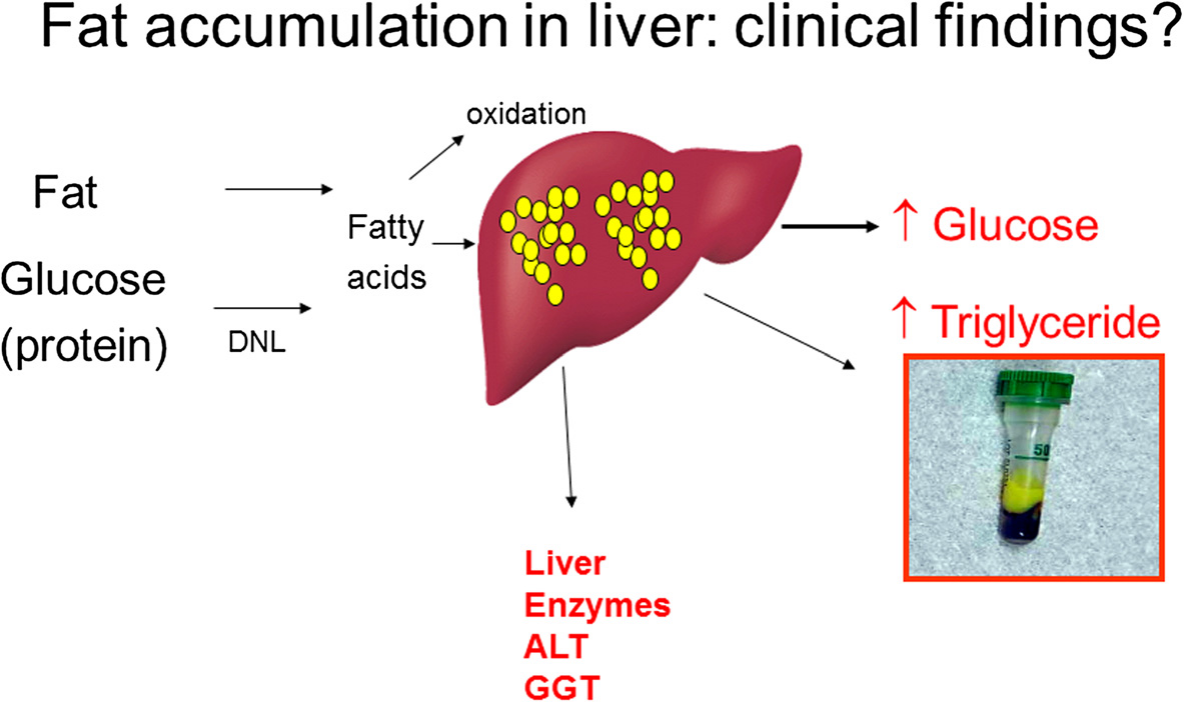
**Biochemical findings supporting excess liver fat.** This simple figure signals the link between excess calories, leading to excess liver fat and the common biochemical findings of high glucose, altered liver enzymes (alanine aminotransferase (ALT) much greater than aspartate aminotransferase (AST)) and hypertriglyceridaemia. When these three biochemical features are present in overweight or obese individuals who are not excessive alcohol drinkers, the likelihood of non-alcoholic fatty liver disease (NAFLD) is high.

Although the above evidence is now well accepted, the exact molecular mechanisms leading to excess hepatic fat accumulation remain elusive. Some authors suggest that hyperinsulinaemia drives hepatic *de novo* lipogenesis whereas others suggest continued excess energy intake, particularly in form of carbohydrate, to be the main factor responsible for hepatic *de novo* lipogenesis and hepatic fat excess [26]. Regardless of the relative contributions of these two alternatives, excess hepatic fat appears to be causally linked to hepatic insulin resistance via a number of potential cellular pathways, including deleterious impaired inflammatory signalling, endoplasmic reticulum stress, excess production of reactive oxygen species, mitochondrial dysfunction, accumulation of triglycerides and/or fatty acyl intermediates, and activation of serine-threonine kinases, as recently reviewed [27]. Recent research has focused in particular on hepatic diacylglycerol (DAG) excess being the main culprit leading to insulin resistance [28]. DAG activates protein kinase Cϵ (PKCϵ), resulting in decreased insulin signalling [28]. Individuals with excess hepatic fat or NAFLD are therefore unable to suppress hepatic gluconeogenesis normally in response to insulin, and the continued excessive hepatic glucose production contributes to hyperglycaemia and development of diabetes. The same individuals also show elevated very low density lipoprotein synthesis and thus elevated circulating triglyceride levels, another example of ‘ectopic’ fat appearing in the circulation. The commonest signal for raised liver fat in such individuals is raised liver enzymes, in particular alanine aminotransferase (much greater than aspartate aminotransferase) and/or γ-glutamyl transpeptidase levels, are recently reviewed [25]. These concepts are demonstrated in Figure 3.

It is thought that in individual destined to develop diabetes, at a given level of peripheral or hepatic insulin resistance, the ability of the pancreas to hypersecrete insulin is overwhelmed, and so frank diabetes ensues. Pancreatic failure is thought to be largely genetically governed, and as discussed before, multiple genes governing insulin secretion have been identified. However, the possibility that excess fat accumulating in pancreas can impair β-cell function is now also emerging [29].

### Is type 2 diabetes reversible via loss of fat or ectopic fat?

The simple answer to this is ‘yes’. If we accept that obesity is causally linked to diabetes and that some individuals can lose considerable weight by surgical or dietary means, diabetes must be reversible. In a recent meta-analysis, Buchwald *et al*. demonstrated that a mean weight loss of around 38.5 kg from a mean pre-surgery BMI of around 48 kg/m^2^ led to diabetes remission in around 78% of patients [30]. Whether some types of bariatric surgery lead to greater diabetes remission for a weight reduction than others remains debated but, suffice to say, the greater the weight loss in general, the greater the chance of T2DM remission. In a more recent meta-analysis [31], patients with a shorter duration of T2DM and lower fasting plasma glucose had higher remission rates, though further randomised studies would be helpful to support and extend these findings; in particular, there was sparse data for ethnic groups outside of white Europeans. Perhaps most impressively, many patients previously on insulin also had T2DM remission [30,31] suggesting that, at least in some patients, pancreatic function previously thought to be completely lost may be recoverable with substantial weight loss.

In terms of diet, the most recent notable study was led by the Newcastle group of Taylor *et al.* [29]. This group demonstrated normalisation of blood glucose in 11 patients with diabetes after 1 week of commencing a 600 kcal/day diet. They also demonstrated improvement in liver fat levels, which fell from around 10% to an average within the normal range at 2.9% by week 8, as well as an improvement in β-cell response, the latter potentially linked to the observed reduction in pancreatic fat [29]. These very impressive findings suggest that a rapid resolution of hyperglycaemia can be achieved by substantial caloric reduction. They also suggest the occurrence of rapid and preferential liver fat loss because hepatic triglyceride content decreased by an average of 30% during week 1 of intervention (*P* < 0.001), whereas total weight reduced by only around 4%. The liver response to insulin also improved by the end of week 1 so that insulin suppression of hepatic glucose output improved from a mean of 43% to 74%, the latter being similar to weight-matched controls without diabetes. These findings all fit with the concept that excess liver fat is intrinsically linked to hepatic insulin resistance and consequently higher fasting glucose levels. Why fat reduction should lead to improved β-cell function is not entirely clear, but is an area in need of greater research.

Of course, these impressive short-term results are merely proof of concept. Cutting energy intake to 600 kcal per day is achievable for most over a short term but, in reality, not sustainable for long periods. The clinical implications of these findings therefore need to further tested in longer-term clinical trials, some of which have now begun. Nevertheless, these observations support the ectopic fat hypothesis for T2DM.

## Summary

In summary, it is clear that obesity is a major risk factor for T2DM, and appears to drive tissue insulin resistance via gain of ectopic fat, with the best-studied organ being the liver. Excess liver fat, and its related condition of NAFLD, is now recognised as a key part of the T2DM puzzle. However, ectopic fat in pancreas may also impair β- cell function, which is an area deserving of more research. It is also clear that different individuals will start to expand their ectopic stores at different BMIs and levels of total adiposity, dependent upon their unmodifiable characteristics (age, sex, ethnicity, family history) so that some groups, such as South Asian men with a family history of T2DM, have a very high diabetes risk even at modest BMIs. Recent genetic data add support to the concept of a diminished subcutaneous capacity in lower BMI individuals being linked to greater metabolic risk [32]. Finally, the definitive evidence of a causal link between obesity and T2DM comes from interventions that can lower weight substantially, either by surgery or diet. Where studied, these studies support a rapid resolution in diabetes linked to a preferential and rapid reduction in liver fat, further supporting the ectopic fat hypothesis for diabetes. The preceding observations are summarised in Table 1. The challenge now is to take advantage of such observations and translate into clinical improvements. Considerable ongoing work is needed to develop such clinical translation, which is crucially important given the rising levels of T2DM worldwide, which will further rise given the continued rise in obesity worldwide [33].

**Table 1:** **Evidence linking obesity to type 2 diabetes**

**Type of evidence**	**Findings**
Epidemiological	BMI is the dominant risk factor for type 2 diabetes. Whereas RRs for CVD with rising BMI tend to be around twofold to threefold once BMI levels reach around 30–35 kg/m^2^, RRs for diabetes are often around a log scale higher, approaching 50 to 80 times at BMI 35 kg/m^2^ versus BMI of 21 kg/m^2^.
Genetic	Genes linked to higher BMI, in particular the *FTO* gene, have been clearly shown to predict T2DM.
Mechanistic	Ectopic fat in key organs that are relevant to glucose metabolism appears to be crucial for tissue insulin resistance. In the liver, ectopic fat via metabolic intermediates interferes with insulin signalling and thereby contributes to higher fasting glucose levels and hypertriglyceridaemia. Characteristics linked to earlier ectopic fat gain include male sex, family history of diabetes and certain ethnic origins.
Clinical	BMI or waist size make up around half of the weighting in diabetes risk scores, regardless of ethnicity. Moreover, around half of all patients with diabetes are obese.
Reversibility	Weight loss via dietary or surgical methods can reverse T2DM and even some patients previously on insulin can show remission, suggesting improvements in β-cell function. Rapid reductions in glucose levels appear to relate to changes in liver fat content in the short term whereas diabetes remission appears to relate to the amount of fat loss in the longer term.

BMI, body mass index; CVD, cardiovascular disease; RR, relative risk; T2DM, type 2 diabetes mellitus.
